# Community-level intimate partner violence and the circumstances of first sex among young women from five African countries

**DOI:** 10.1186/1742-4755-7-11

**Published:** 2010-06-19

**Authors:** Anu Manchikanti Gómez, Ilene S Speizer

**Affiliations:** 1 Department of Maternal and Child Health, Gillings School of Global Public Health, University of North Carolina at Chapel Hill, Chapel Hill, NC, USA; 2 Carolina Population Center, University of North Carolina at Chapel Hill, Chapel Hill, NC, USA

## Abstract

**Background:**

Gender-based violence is an important risk factor for adverse reproductive health (RH). Community-level violence may inhibit young women's ability to engage in safer sexual behaviors due to a lack of control over sexual encounters. Few studies examine violence as a contextual risk factor.

**Methods:**

Using nationally representative data from five African countries, the association between community-level physical or sexual intimate partner violence (IPV) and the circumstances of first sex (premarital or marital) among young women (ages 20-29) was examined.

**Results:**

In Mali, and Kenya bivariate analyses showed that young women who had premarital first sex were from communities where a significantly higher percentage of women reported IPV experience compared to young women who had marital first sex. Multivariate analyses confirmed the findings for these two countries; young women from communities with higher IPV were significantly more likely to have had premarital first sex compared to first sex in union. In Liberia, community-level IPV was associated with a lower risk of premarital sex as compared to first sex in union at a marginal significance level. There was no significant relationship between community-level IPV and the circumstances of first sex in the Democratic Republic of Congo or Zimbabwe.

**Conclusion:**

These findings indicate that context matters for RH. Individualized efforts to improve RH may be limited in their effectiveness if they do not acknowledge the context of young women's lives. Programs should target prevention of violence to improve RH outcomes of youth.

## Introduction

Gender-based violence is an important risk factor for adverse reproductive health (RH) outcomes for women throughout the world. Specifically, intimate partner violence (IPV) has been linked to increased risk of human immunodeficiency virus (HIV), sexually transmitted infections (STIs), unintended pregnancy and pregnancy loss [1-13]. IPV is an important public health concern in sub-Saharan Africa, where between 13% and 54% of women report experiencing IPV during their lifetimes [8,14,15].

Nearly half of new HIV infections are among youth ages 15-24 [16]. In sub-Saharan Africa, women ages 15-24 are three times more likely to be infected with HIV than their male peers [17]. Young women may especially be at risk of experiencing IPV due to power imbalances with older partners [18,19]. One strategy for HIV prevention is promoting delayed sexual debut among youth. Early sexual debut is associated with increased likelihood of risky sexual behavior later in life, lower levels of knowledge of STI and HIV prevention, and decreased ability to negotiate condom use during sex [20-23]. Furthermore, early sexual debut may contribute to greater lifelong risks of poor RH, including HIV and STIs, due to longer periods of sexual activity and more lifetime sexual partners [20,22]. Conversely, women who engage in early and premarital sex may be at reduced risk of HIV because they may be better able to refuse sex without a condom as compared to their married counterparts, who may face more difficulty negotiating condom use with their husbands [24]. Premarital sex is of particular interest in this region, where much attention has been focused on abstinence education to reduce the spread of HIV. The underlying assumption of abstinence education is that youth control the circumstances of first sex, ignoring the possibility that first sex is unwanted or coerced, or that young women may be unable to negotiate condom use due to power imbalances with older partners [25,26].

The majority of research examining linkages between IPV and RH focuses on individual-level risk factors. An ecological approach also considers community-level influences [27]. While there have been calls by numerous researchers for interventions and programs that consider the context of women's lives, particularly factors such as gender, culture, and social norms [27-29], little research has taken this approach and examined RH outcomes in the context of communities where violence is predominant. In particular, the fear or experience of violence may limit women's ability to negotiate safe sex behaviors, including condom use, sexual initiation, and monogamy [30-33].

Specific contextual factors, including community attitudes toward gender roles and wife beating and levels of socioeconomic status and literacy, may influence the likelihood of IPV and RH outcomes [30,34-38]. However, most research from sub-Saharan Africa focuses on individual-level determinants of HIV risk behavior among youth and does not consider violence as a contextual risk factor for poor RH. The present study explores the effect of community-level IPV on the circumstances of first sex among sexually experienced women ages 20-29 in five sub-Saharan African countries. Utilizing an ecological approach to studying the circumstances of first sex, we test the hypothesis that young women living in communities with higher levels of physical or sexual IPV are more likely to have experienced premarital first sex as compared to young women from communities with lower levels of IPV. In communities where violence is more prevalent, young women may be more likely to report premarital sex because they experienced a forced or coerced sexual debut prior to marriage. Moreover, even if first sex was not forced, young women from more violent communities may be less likely to decline premarital sexual advances because they fear that they will become a victim of IPV due to the normalization of violence in their communities. By focusing on 20-29-year-old women, we examine a recent cohort of young women who have completed adolescence, the majority of whom were sexually experienced and have entered a first union.

## Methods

This secondary data analysis utilized recent Demographic and Health Surveys (DHS) for five sub-Saharan African countries: Liberia (2007), Zimbabwe (2005/06), Mali (2006), the Democratic Republic of Congo (DRC) (2007), and Kenya (2003).

### Setting

The countries included in this study represent a variety of geographic regions in Africa, including Liberia and Mali in West Africa, DRC in Central Africa, Zimbabwe in Southern Africa, and Kenya in East Africa. In each of the countries, the majority of households are located in rural areas [39]. Levels of fertility are generally high, with the total fertility rate ranging from 3.8 in Zimbabwe in 2005/6 to 6.6 in Mali in 2006 [39]. HIV is endemic in Kenya and Zimbabwe, with a lower prevalence in the other three countries [40]. While a number of the countries have enacted legislation to combat violence against women, IPV continues to be a serious social problem [41,42].

### Data

These five countries were selected because they had recent data and included the domestic violence module, which collected information from women about IPV experiences. The DHS are nationally representative surveys of women ages 15-49 and, in some countries, men ages 15-59 in Africa, Latin America, and Asia. Multi-stage, probability sampling is used to identify households. In the datasets utilized here, the domestic violence module was administered to one woman in each household; however, only ever-married women were asked about IPV experience. Women were only administered the domestic violence module if the interviewer could assure the privacy of the woman. The unweighted number of women surveyed using the domestic violence module ranges from 4,913 in Liberia to 9,849 in Mali. Because the focus of this study is on the circumstances of first sex, the present analysis examined sexually experienced women ages 20-29. Across the countries, the majority of women in this age group were sexually experienced, ranging from 89% in Zimbabwe to 99% in Liberia. Based on the selection criteria, the sample of eligible women ranged from 2,480 sexually experienced women ages 20-29 in Liberia to 5,206 in Mali (unweighted). Additionally, this analysis examines the role of community-level IPV on the circumstances of first sex, controlling for a woman's own IPV experience. To perform this analysis, only women currently in union who were asked the IPV questions were included; this reduces the sample sizes significantly (unweighted sample sizes vary from 1,341 in Liberia to 3,772 in Mali). Notably, by focusing on the sample of women who are most recently in union and aged 20-29 years, the sample included is more educated, more likely to be from urban areas and to be in union, and more likely to be exposed to the radio or television. The differences between the 20-29 year old women and their non-included surveyed counterparts were statistically significant in most countries, with the exception of in the Congo, where only education level and union status were significant, and Liberia, where only education level was significant. By focusing on this recently in union sample across the multiple countries, the same young adult bias is introduced into all analyses, and we are able to make recommendations for future generations of youth who will be attaining marriageable age in the near future.

### Variables

The outcome variable had two categories that reflect the circumstances of sexual debut: premarital first sex and first sex in union. Being in union was defined as being formally married or cohabiting with a partner. The outcome variable was created based on the women's report of their age at first sex, union status and age at first union questions.

The key independent variable of interest was the prevalence of lifetime sexual or physical IPV in each woman's community. For the purposes of this analysis, the community was defined based on the lowest level of sampling, the primary sampling unit (PSU), used in each country. In selected PSUs, or clusters, typically 20-30 households are sampled and all women of reproductive age are surveyed. At the individual level, women were considered to have ever been victims of sexual or physical IPV during their lifetimes if they reported any of the following experiences with a husband or partner: being pushed, shaken, or having something thrown at them; being slapped; having their arm twisted or hair pulled; being punched with a fist or something that could harm them; being kicked, dragged, or beaten up; attempted choking or burning; being threatened or attacked with a knife, gun, or any other weapon; being physically forced to have sex when not desired; or being forced to perform any sexual act when not desired. For each cluster, the weighted mean level of sexual or physical IPV was calculated using weights designed for the domestic violence sample. To permit enough variability within communities in responses to the IPV questions, this analysis only included clusters where 10 or more women were administered the domestic violence module. A small number of clusters (and thus individual-level observations from those clusters) were dropped because too few women were asked about IPV. DRC had the most observations dropped because of small clusters (13%, unweighted); in the remaining countries less than 4% of observations were dropped because of small cluster size. The unweighted analysis sample sizes for this study vary from 2,463 sexually experienced young women in Liberia to 5,032 in Mali.

Models were also run to examine the role of community-level IPV on the circumstances of first sex, controlling for a woman's own IPV experience. To perform this analysis, only women currently in union who were asked the IPV questions were included; this reduced the sample sizes significantly (unweighted sample sizes vary from 1,341 in Liberia to 3,772 in Mali).

Control variables in all models included categorical indicators of age (20-24 or 25-29 years), place of residence (urban or rural), highest education level (no education, primary, secondary, or higher), and exposure to radio and television (exposed almost daily or not at all, less than once a week, at least once a week). Additionally, the DHS wealth quintile was included as a proxy measure of socioeconomic status [43]. These control variables were selected based on previous research that highlight their importance to the RH of youth [44-54].

### Analytic Approach

Descriptive statistics were calculated for each country. In a bivariate analysis the mean community-level of IPV was computed by the circumstances of first sex to determine whether women who experienced premarital first sex were more likely to come from communities with a higher prevalence of IPV as compared to women who experienced first sex in union. Adjusted Wald tests were used to compare differences in means. For multivariate analyses, logistic regression was performed for the binary outcome variable. Adjusted odds ratios and 95% confidence intervals are presented. Model 1 of the multivariate analyses determined whether, controlling for demographic variables, women living in communities with higher IPV were more likely to have had premarital first sex as compared to women from communities with a lower prevalence of IPV. To examine the effect of community-level IPV on the circumstances of first sex including a woman's own IPV experience, two additional models were performed. In Model 2, a logistic regression model comparing premarital first sex to first sex in union for currently in union women was performed to determine the role of community-level IPV in the reduced analysis sample that includes only women who were asked the partner violence questions. Additionally, Model 3 builds on Model 2 by examining an individual woman's own IPV experience in relation to the circumstances of first sex. All analyses included the control variables listed above, adjusted for the sample design features of the DHS, and included sample weights by employing survey commands in Stata/SE, version 10.1 software. This secondary data analysis project was reviewed by the University of North Carolina Institutional Review Board and declared exempt.

## Results

Between 36 and 39% of all female respondents surveyed in the five countries were aged 20 to 29 (not shown). In each country, there was a greater proportion of 20-24-year-olds than 25-29-year-olds (see Table 1). The proportion of young women ever in union varied from 72% in Liberia to 99% in Mali. The majority of young women ages 20-29 had sexually debuted by the time of the survey, ranging from 89% in Zimbabwe and 90% in Kenya to 99% in Liberia (not shown). There was considerable variation in the circumstances of first sex across the five countries. While the majority of young women had first sex in union in Zimbabwe (63%) and Mali (77%), in the other three countries, a greater percentage of young women had premarital first sex than first sex in union; the highest levels of premarital first sex were in Liberia (68%) and Kenya (67%).

**Table 1:** Descriptive statistics for young women (ages 20-29) from five African countries

	Liberia, 2007	Zimbabwe, 2005/06	Mali, 2006	DRC, 2007	Kenya, 2003
**Univariate:**					
Young adult sample (unweighted sample size)	n = 2463	n = 2916	n = 5032	n = 3171	n = 2650
20-24	53.8	53.4	50.5	56.2	52.0
25-29	46.2	46.6	49.5	43.8	48.0
% of young adults ever in union	72.0	89.8	98.7	84.0	81.0
					
					
Young adult circumstances of first sex					
Premarital first sex	67.5	36.6	23.2	58.5	66.7
First sex in union	32.5	63.4	76.8	41.6	33.3
					
Community mean lifetime sexual or physical IPV victimization (range)	0.37 (0.0-0.9)	0.27 (0.0-0.8)	0.21 (0.0-0.8)	0.64 (0.0-1.0)	0.43 (0.0-1.0)
					
**Bivariate:**					
Community mean level lifetime sexual or physical IPV by circumstances of first sex:					
Premarital first sex	0.376	0.27	0.237	0.631	0.441
First sex in union	0.385	0.286†	0.206***	0.635	0.406*

Notes: Eligible sample is ever sexually experienced women residing in clusters where 10 or more women answered the domestic violence module. A small number of women were dropped due to cluster size < 10. The weighted percentage of women dropped in each country is: Liberia - 2.2%; Zimbabwe - 2.4%; Mali - 0.5%; DRC - 10.8%; Kenya - 3.6%. All frequencies and means are weighted. Adjusted Wald test used to compare differences in means. † p ≤ 0.10; * p ≤ 0.05; ** p ≤ 0.01; *** p ≤ 0.001.

The percentage of women of all ages who reported ever experiencing sexual or physical IPV is also presented in Table 1 as a weighted community mean. The mean value ranged from 0.21 (or 21%) in Mali to 0.64 (or 64%) in DRC. In DRC and Kenya, the range of the mean of community-level IPV varies from zero to one, indicating that in some communities (clusters), no women experienced IPV whereas in other communities, all women reported IPV experience. The other three countries also included communities where no women reported lifetime sexual or physical IPV, and other communities where up to 80% or 90% of women reported lifetime IPV experience. Notably, the highest prevalence of IPV experience was in DRC, where 64% (community mean of 0.64) of women reported having experienced IPV. This may be indicative of DRC being a more violent environment overall as well as for women.

At the bottom of Table 1, the mean community-level physical or sexual IPV is presented by the female youth's circumstances of first sex. In Mali, and Kenya, community-level IPV was significantly different by the circumstances of first sex in the expected direction. For example, in Mali, women who experienced premarital first sex came from communities with a higher prevalence of IPV (23.7%) as compared to women who had first sex in union who were from communities with a lower prevalence (20.6%). The same pattern was found in Kenya. A significant difference in community prevalence of IPV was found in Zimbabwe; however, the effect was the opposite: women who had first sex in union were from communities with a higher prevalence of IPV (28.6%) than women who had premarital first sex (27.0%). In Liberia and DRC, there were no significant differences in the mean level of community IPV by the first sex category; however, in DRC, community-level IPV was notably higher among young women who had premarital first sex and women who had first sex in union as compared to the other four countries.

Multivariate analyses of the association between community-level IPV and whether first sex was premarital or in union are presented in Table 2. Three models are presented. In Model 1, the full sample of sexually experienced young women was included, controlling for the key demographic variables. In Model 2, the same analysis was performed, but only including those young women who were in union and thus answered the IPV questions; this is a subset of the full analysis sample. Finally, Model 3 used the reduced sample and included the respondent's own experience of IPV to determine if community-level IPV prevalence is associated with first sex being premarital or in union, controlling for a woman's own exposure to IPV. In Mali and Kenya, we find the hypothesized effect in all models. Young women who were from communities with a higher level of sexual or physical IPV were significantly more likely to have had premarital first sex than first sex in union. This was true also in the model that controlled for the woman's own IPV experience (Model 3) with the reduced analysis sample. Conversely, in Liberia, we find that women who were from communities with a higher prevalence of IPV were significantly less likely to have had premarital first sex and thus more likely to have had first sex in union (Model 1); this is counter to the hypothesis being tested. In Models 2 and 3 with the reduced analysis sample, these effects were attenuated. In Zimbabwe and DRC, there was no association of community-level IPV on whether first sex was premarital or in union. Finally, it is interesting to note that only in Kenya was there a borderline association between a woman's own IPV experience and the circumstances of first sex such that women who have ever experienced IPV were somewhat more likely to have had first sex as premarital rather than in union.

**Table 2:** Multivariate logistic regression odds ratios (confidence intervals) examining the effect of community mean sexual or physical IPV on whether first sex was premarital or in union among young women (ages 20-29) by country

	Premarital vs. First sex in union Model 1^a ^(full sample)	Premarital vs. first sex in union Model 2^b ^(IPV sample; currently in union)	Premarital vs. first sex in union Model 3^c ^(IPV sample; currently in union)
**Liberia, 2007**			
Community mean IPV	0.55 (0.30-1.00)*	0.59 (0.29-1.19)	0.47 (0.22-1.01) †
Own IPV experience (Yes vs. no)	NA	NA	1.25 (0.87-1.79)
**Zimbabwe, 2005/06**			
Community mean IPV	0.65 (0.34-1.23)	1.24 (0.62-2.49)	1.08 (0.52-2.27)
Own IPV experience (Yes vs. no)	NA	NA	1.14 (0.87-1.48)
**Mali, 2006**			
Community mean IPV	4.50 (2.29-8.84)***	5.60 (2.62-11.97)***	4.30 (1.93-9.56)***
Own IPV experience (Yes vs. no)	NA	NA	1.29 (0.92-1.79)
**DRC, 2007**			
Community mean IPV	1.01 (0.54-1.89)	0.98 (0.43-2.21)	0.88 (0.37-2.09)
Own IPV experience (Yes vs. no)	NA	NA	1.11 (0.75-1.64)
**Kenya, 2003**			
Community mean IPV	1.82 (1.05-3.15)*	2.82 (1.49-5.32)***	2.30 (1.15-4.61)*
Own IPV experience (Yes vs. no)	NA	NA	1.24 (0.97-1.58)†

Notes: Reference group is first sex in union. All models controlled for age, urban residence, educational status, wealth quintile and exposure to the radio and television. † p ≤ 0.10; * p ≤ 0.05; ** p ≤ 0.01; *** p ≤ 0.001. Models 2 and 3 use domestic violence weights including only women currently in union who responded to the question on IPV experience. Unweighted sample sizes for IPV samples: Liberia - 1341; Zimbabwe - 2068; Mali - 3772; DRC - 1036; Kenya - 1776. ^a^Model 1, full sample; ^b^Models 2 with reduced domestic violence sample; ^c^Model 3 with reduced domestic violence sample and inclusion of women's own IPV experience.

## Discussion

This study examined the hypothesis that young women living in communities with higher levels of IPV are more likely to have premarital first sex rather than first sex in union. Support was found for this hypothesis in Mali and Kenya. In particular, in these countries, young women living in communities with higher mean levels of sexual or physical IPV were at increased risk of having engaged in premarital sex compared to women from communities with lower IPV prevalence. In Liberia, the opposite effect was found, with residence in more violent communities associated with a lower risk of premarital first sex. Community-level IPV did not appear to have an effect on the circumstances of first sex in Zimbabwe and DRC.

Unlike many studies examining sexual behavior among young people in sub-Saharan Africa, this analysis incorporates violence as a contextual risk factor. Violence is an important risk factor for HIV and other adverse RH outcomes, fostering environments that make it difficult for women to reduce their own HIV risk due to lack of control over sexual encounters. An additional strength of this study is the usage of nationally representative datasets that include the same measures of IPV, first sexual experience, and socio-demographic variables, as well as utilizing standardized training and data collection procedures. Finally, by including 20- to 29-year-olds as the population of interest, we are able to examine a cohort of young women who have nearly all sexually debuted and thus reduce bias in the results compared to typical analyses that focus on 15- to 24-year-olds, especially since many of the younger women are not yet sexually experienced.

A number of limitations must be acknowledged. First, this analysis reflects a diverse range of countries, including countries with low and high HIV prevalence, from varying regions of Africa, and that are Francophone and Anglophone. These countries were included because they were the only ones that met the criterion of having a domestic violence module and having the overwhelming majority of women living in a cluster where at least 10 women answered the domestic violence module. However, the diversity of included countries renders the creation of programmatic recommendations difficult, particularly since violence may be deeply embedded in cultural and social norms. Second, 10 women from each cluster may not accurately reflect a community's experience with IPV. Moreover DHS data are not representative at the cluster level, and clusters may not reflect natural communities. Third, the community where a young woman currently resides may not be the same community where she sexually debuted. Additionally, violence behaviors may have changed over time, and the current level of IPV may not be reflective of the environment that women faced at the time of their sexual debut. Fourth, a woman's current IPV experience may have happened in the recent past and may be associated with the circumstances of first sex but with the opposite direction of causality; women who had premarital first sex may be more likely to experience IPV than women who had first sex in union. This variable was included to permit control of a woman's own IPV experiences and to assess the effect of contextual influences above and beyond the woman's own experiences with IPV. Finally, these cross-sectional data did not permit us to explore the specific mechanism by which community-level IPV and the circumstances of sexual debut are linked; this requires more detailed data collection, possibly using qualitative or longitudinal survey data.

To our knowledge, this is the first study to examine community-level IPV in relation to the circumstances of first sex among young women. One other study has examined the role of spousal abuse norms on early and premarital sexual debut and found that higher community support of wife beating was associated with earlier and premarital sex in Kenya and Tanzania; however, the findings were in the opposite direction in Zimbabwe and Malawi [55]. The results of our analysis support findings from other population-based research that find that context matters to RH outcomes, particularly gender inequality [30,38]. IPV is a severe expression of gender inequality in a society and understanding if and how it influences health outcomes has important programmatic implications.

Support for our hypothesis was found in only two countries, and there was no statistically significant relationship between community-level IPV and circumstances of first sex in DRC, the country with the highest mean level of IPV. DRC differs from other countries in this study due to its post-conflict status and the ongoing violations of women's human rights, with rape used as a tool of war [56]. These results may indicate that violence is more endemic in this society, and there is less variability across communities. Additionally, in Liberia, an unexpected result was noted, with higher mean levels of IPV being associated with less premarital first sex experience. Future research in this setting, particularly studies utilizing qualitative research methods, may shed light on these findings and whether certain protective cultural factors are present in communities with higher levels of IPV, including community-level norms around other aspects of gender inequality not examined in this study.

We hypothesized that young women living in communities with higher levels of IPV may be more likely to engage in premarital first sex due to greater exposure to forced sex or the fear or threat of violence. In countries with high levels of HIV, programs should target communities with higher levels of violence for HIV prevention efforts, as well as implement violence prevention programs in general. The findings of this and other studies indicate that context matters for RH. Individualized interventions to reduce the risk of HIV, STIs and unintended pregnancy may be limited in their effectiveness if they do not consider the context of young women's lives in their efforts.

## Competing interests

The authors declare that they have no competing interests.

## Authors' contributions

AMG undertook the data coding and analysis and developed the first draft of the manuscript. ISS conceived of the study and finalized data analysis for the submitted manuscript. Both authors read and approved the final manuscript.
